# Preliminary Assessment of Mycobiome at Former Quarry Site That Hosts a Diverse and Abundant Orchid Population

**DOI:** 10.3390/microorganisms13102390

**Published:** 2025-10-17

**Authors:** Viswambharan Sarasan, Dean Williams, Zoe Ringwood

**Affiliations:** 1 Royal Botanic Gardens, Kew, Surrey TW9 3DS, UK; 2 Essex Wildlife Trust, Abbotts Hall, Maldon Road, Great Wigborough, Colchester CO5 7RZ, UKzoer@essexwt.org.uk (Z.R.)

**Keywords:** orchid mycorrhiza, chalk grassland restoration, orchid colonisation, soil fungal communities, calcareous grassland, ecological restoration

## Abstract

Former quarries offer unique opportunities for biodiversity restoration, yet their potential for orchid meadow creation remains underexplored. This study screened soils to study whether these habitats harbour key orchid-compatible fungi essential for orchid colonisation. We conducted comparative analyses of fungal community composition across restored quarry sites using alpha and beta diversity metrics, NMDS ordinations, and regression models linking orchid abundance with fungal diversity. Using soil metabarcoding across four restored sites, the results showed that orchid abundance strongly correlated with fungal diversity, including mycorrhizal families such as Sebacinaceae and Thelephoraceae. The gorge-based site supported the highest orchid density and richest fungal assemblage. These findings demonstrate that former quarries can sustain the fungal networks required for orchid recruitment, providing a foundation for large-scale restoration strategies. Association analysis revealed that orchid abundance, though on a limited scale, is a strong predictor of fungal diversity, indicating that denser orchid populations support richer fungal communities. Despite its limited scale, this study demonstrates that former quarries can provide both the physical conditions and the fungal networks necessary for orchid establishment, offering a practical model for restoring orchid-rich meadows and enhancing biodiversity in former quarries.

## 1. Introduction

Disused quarry sites serve an important role in wildlife conservation, although natural regeneration is very slow compared with the rate of land use in modern quarrying [1]. Spontaneously restored post-mining sites exhibited similar species richness compared to technically reclaimed sites; however, they supported a greater number of rare species [2]. The open early successional habitats of disused quarry sites can provide an important habitat for species of orchid [3,4].

The spatial distribution of soil fungi is critical in determining the spatial patterns of plant species [5]. Orchidaceae, with approximately 28,000 [(https://www.kew.org/read-and-watch/orchid-family-tree), accessed on 15 July 2025] described species and new ones being described on a continuous basis, is one of the largest families of flowering plants globally. The natural recruitment of orchid seedlings in the wild is dependent on mycorrhizal fungi for their survival and establishment [6,7,8,9]. Distribution and relative abundance of orchid mycorrhizal fungi (OMFs) in soil is a driver for successful colonisation of orchids at the landscape level, with sites with thriving orchid populations often characterized by a high abundance of these fungi [9,10,11,12]. However, detection of certain mycorrhizal fungi is especially difficult in soil beyond the rhizosphere [13].

Chalk and limestone quarries have been recognized as vital habitats for wildlife, promoting the establishment of diverse and species-rich communities throughout various regions of the United Kingdom [1]. A comprehensive study of 42 European cities revealed the presence of 70 orchid species thriving in man-made environments, primarily in sand and clay pits [14]. Of the four species of *Orchis* studied under severe climate change scenarios, a marked contraction in their distribution at the warmer edges of their ranges was observed [15]. These findings suggest that moderate climate warming may benefit the studied orchid species by facilitating their geographic expansion.

This novel study assesses the soil fungal microbiome in chalk grassland habitat at different stages of establishment within a former quarry site that is now a nature reserve. It analyses the relationships between key fungal groups and orchids, and discusses in detail whether disused quarries can be transformed into orchid-rich meadows through targeted restoration. The premise is that the soil in these habitats already harbour key mycorrhizal fungi essential for orchid colonisation.

## 2. Materials and Methods

### 2.1. Site Description

The study area is Grays Gorge, part of Essex Wildlife Trust’s Chafford Gorges Nature Reserve, located within the town of Chafford Hundred in the south of Essex. The area was extensively quarried for brick earth, gravel, chalk, and flint. Clays were quarried and combined with chalk to create Portland Cement from 1874. The quarrying created the gorges that form much of the nature reserve, an area rich in biodiversity and geological history.

Quarrying ceased at Grays Gorge in the 1920s, and it has been managed by Essex Wildlife Trust since 1987. Today, the gorge is protected as a Site of Special Scientific Interest (Grays and Thurrock Chalk Pit SSSI) for its rare invertebrate assemblages supported by the chalk grassland habitat and ephemeral pools. The chalk grassland areas are also important for their botanical interest, especially their abundance and diversity of orchids. A total of eight orchid species has been recorded at the site, including the nationally scarce Man orchid—*Orchis anthropophora*.

Some of the grassland areas had succeeded to scrub and woodland, due to being unmanaged in the past. Essex Wildlife Trust is now restoring these areas through active management and scraping the soil back to the chalk. The habitats at the site today include chalk grassland, bare chalk, scrub, woodland, and lakes and ponds. The gorge is nestled within an urban area and is flanked by roads and residential areas. It is a popular spot for dog walkers and general recreation.

### 2.2. Study Sites

**Figure 1:**
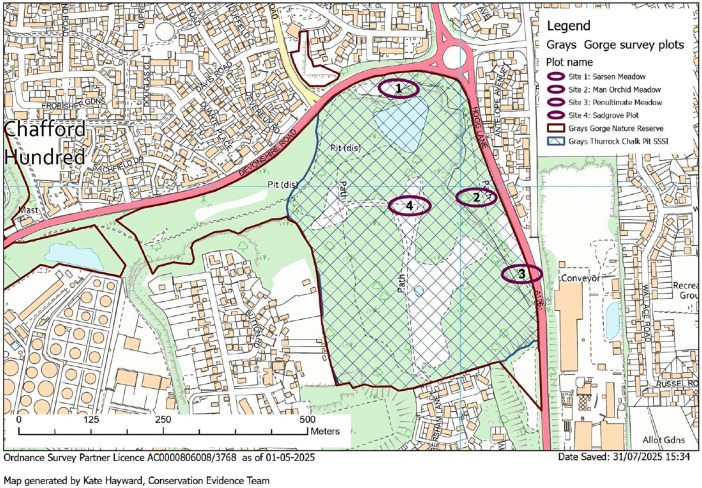
Map of Grays Gorge, Chafford Hundred, Essex, showing location of four study sites.

The study focussed on four sites within Grays Gorge (Figure 1). Three sites (Sites 1, 2, and 3) were located at the top of the gorge, the top shelf near the boundary of the site, and the adjacent road. The fourth site (Site 4) was located at the bottom of the gorge.

Over a century ago, a manmade table was constructed around the top of the gorge (the ‘top shelf’) by bringing soil from the surrounding areas, and the gorge sites were subsequently restored. Today, the general conservation management of the four study sites aims to cut and clear vegetation on rotation to remove nutrients and maintain an open habitat.

Site 1: Sarsen Meadow

This area is located on the top shelf of the gorge and supports a number of orchid species, including a good number of *Orchis anthropophora* (Man Orchid), *Anancamptis pyramidalis* (Pyramidal Orchid), and *Neottea ovata* (Common Twayblade). It has relatively dense vegetation and some scattered scrub. The understorey vegetation is cut biennially in the early autumn and the arisings are collected and removed.

Site 2: Man Orchid Meadow

This meadow supports an important population of *O. anthropophora*. It is located on the top shelf of the gorge and is cut biennially in early autumn (removing all arisings) and on rotation with Sarsen Meadow, to ensure areas there are areas left unmanaged each year. Some of the scrub has been coppiced to open up the area.

Site 3: Penultimate Meadow

This area was heavily overgrown with dense scrub, shrubs, birch saplings, and various other plant species. The *O. anthropophora* populations at this location were found on a slope with chalky soil. This area is cut annually in early autumn, removing arisings. Works have been in progress to open up this area over the past 10 years.

Site 4: Sadgrove Plot

This site is located inside the gorge, at the base. The topsoil was scraped back to chalk over 10 years ago and the chalk layer is still clearly visible. Vegetation has slowly colonised to create an open meadow with limited vegetation. New populations of *O. anthropophora*, *Dactylorhiza fuchsii* (Common-spotted orchid), and *N. ovata* have appeared in recent years. Orchid abundance and diversity is high at this site. For management, half of the area is cut on rotation each year, removing all arisings.

### 2.3. DNA Extraction and Purification

Four soil samples were collected from all four study sites using a belt transect method. Samples were taken at 1.5-m intervals along the entire length of the meadow, at a depth of approximately 10 cm. Each single sample was a collection of pooled three sub-samples to obtain a wider representation of fungal diversity. DNA was extracted and purified from soil samples stored in BashingBead™ Lysis Tubes (Cambridge Bioscience, Cambridge, UK) using the Quick-DNA™ Fecal/Soil Microbe Miniprep Kit following the manufacturer’s instructions. Soil samples were disrupted using a tissue lyser (QIAGEN, Manchester, UK) for approximately 3 min at 25 Hz. The disrupted mixture was centrifuged, and the supernatant was carefully transferred to Zymo-Spin™ III-F Filter Tubes (Zymo Research, Irvine, CA, USA). Genomic lysis buffer was added to the filtrate. The resulting solution was passed through a Zymo-Spin™ IICR Column (Zymo Research, Irvine, CA, USA) to bind DNA. The column was cleaned using the DNA Pre-Wash Buffer followed by the g-DNA Wash Buffer to remove impurities. Purified DNA was eluted and passed through a pre-prepped Zymo-Spin™ III-HRC Filter (Zymo Research, Irvine, CA, USA) for final purification. The concentration and quality of the DNA from all 20 eluted samples were measured using a Nanodrop 2000/2000c Spectrophotometer (Thermo Scientific, Waltham, MA, USA). Purified DNA samples were amplified by PCR in the internal transcribed spacer 2 (ITS2) region, targeting fungi as part of the eDNA survey pipeline. Three replicate PCRs per sample were performed using primers described by White [16]. Amplification success was confirmed via gel electrophoresis. PCR replicates were pooled and purified, and sequencing adapters were added, with confirmation by gel electrophoresis.

### 2.4. Sequencing and Initial Data Processing

Sequences were quantified using a Qubit Broad Range Kit (ThermoFisher, Altrincham, UK) as per the manufacturer’s protocol. The final library was sequenced using an Illumina MiSeq V3 kit (Illumina, San Diego, CA, USA) at 10.5 pM with a 20% PhiX spike-in. Resulting sequence data were processed using a specialized bioinformatics pipeline. This involved filtering and trimming data, merging paired ends, eliminating sequencing errors (e.g., chimeras), clustering similar sequences into molecular operational taxonomic units (OTUs), and aligning representative sequences from each cluster with a reference database. These steps converted raw sequence data into usable datasets for ecological analysis.

Sequences were demultiplexed based on the combination of i5 and i7 index tags using bcl2fastq (v2.20.0.422 https://support.illumina.com/). Paired-end FASTQ reads were merged with USEARCH v11, requiring a minimum overlap of 80% of the total read length. Quality filtering retained sequences with an expected error rate per base of 0.01 or below. Sequences were dereplicated by sample, retaining singletons. ITSx (v1.1b1) was used to extract fungal ITS2 sequences, removing primers and any remaining ribosomal sequences.

Unique ITS2 sequences were denoised using UNOISE, retaining zero-radius OTUs (ZOTUs) with a minimum abundance of eight in at least one sample. Taxonomic assignments were made through sequence similarity searches against two reference databases: NCBI nucleotide database (NCBI nt; downloaded 28 September 2021) and UNITE (v8.2). Hits required a minimum e-score of 1 × 10^−20^ and coverage of at least 90% of the query sequence. Consensus taxonomic assignments were made at the lowest possible taxonomic level, ensuring consistency in matches. Conflicts were resolved manually. Minimum similarity thresholds of 98%, 95%, and 92% were applied for species-, genus-, and higher-level assignments, respectively.

### 2.5. OTU Table and Abundance Thresholds

The OTU table was filtered to exclude low-abundance OTUs from each sample using thresholds of <0.025% or <10 reads, whichever was greater. Unidentified, non-target, and common contaminants (e.g., human and livestock DNA) were eliminated. Identifications were aligned with the GBIF taxonomic backbone (3 March 2021 edition; downloaded from https://hosted-datasets.gbif.org/datasets/backbone/2021-03-03/ (accessed on 28 September 2021)) to combine results from different databases. Assignments based on fewer than three hits were flagged as tentative.

### 2.6. OTU Clustering and Normalization

ZOTUs were clustered at a 97% similarity threshold using USEARCH, and an OTU-by-sample table was generated by mapping dereplicated reads to representative OTUs at the same threshold. Low-abundance OTUs were removed, and relative abundances were calculated by normalizing each sample column to 100%.

DNA metabarcoding followed the pipeline as detailed by Dove et al. [17], targeting the ITS2 region using primers from White [16]. Triplicate PCRs were performed per sample, and DNA was quantified using a Qubit kit. Sequencing was conducted on an Illumina MiSeq platform with a 20% PhiX spike-in.

Sequence processing included demultiplexing with bcl2fastq (v2.20.0.422), merging paired-end reads with USEARCH v11, and extracting ITS2 sequences using ITSx. Denoising was performed with UNOISE to generate ZOTUs. Taxonomic assignments were made using NCBI nt and UNITE (v8.2), applying similarity thresholds of 98%, 95%, and 92% for species, genus, and higher taxonomic levels, respectively [18,19,20,21].

Limitations include potential misidentification due to incomplete reference databases, low-quality DNA, or environmental contaminants. In ambiguous cases, higher-level or multiple identifications were provided.

ZOTUs were clustered using a 97% similarity threshold with the USEARCH tool. An OTU-by-sample table was generated by mapping dereplicated reads for each sample to representative OTU sequences, with an identity threshold of 97%. Low-abundance detections were removed. Filtered OTU table values were expressed as percentages, with each sample column summing to 100% to represent the relative abundance of OTUs within samples.

### 2.7. Statistical Analysis

All analyses were conducted using R software version 4.4.2. To assess and visualize the alpha diversity of fungal occurrence at each site, the number of operational taxonomic units (OTUs) and their relative abundances were utilized to calculate the Shannon index, Simpson index, and species richness index. The analyses were conducted using the R packages ‘dplyr’, ‘tidyr’, ‘stringr’, and ‘ggplot2’. Subsequently, Shannon index values for each site were statistically compared using the Kruskal–Wallis non-parametric test, followed by Dunn’s post hoc test and Benjamini–Hochberg correction for multiple comparisons.

Beta diversity and similarity patterns among the different sites were evaluated by calculating the Bray–Curtis dissimilarity matrix, based on the relative abundances of Basidiomycota. This was accomplished using the ‘vegdist’ function from the ‘vegan’ package. The resulting coefficients were then used to generate a non-metric multidimensional scaling (NMDS) plot and to calculate the corresponding 3D stress values. To determine whether fungal communities differed significantly among sites, a permutational multivariate analysis of variance (PERMANOVA) was performed, with robustness tested through 999 permutations using the ‘adonis’ function from the ‘vegan’ package in R [22].

The distribution of Basidiomycota OTUs across sites was further compared by creating a presence/absence matrix, wherein relative abundances were converted to binary values (0 for 0% and 1 for >0%). Only OTUs identified to the Order level were retained for this analysis. The presence/absence data for each subsite were summed to indicate the frequency of detection for each OTU at a given site, resulting in values ranging from 0 to 4. These data were visualized using R packages such as ‘tidyverse’, ‘ggplot2’, and ‘viridis’ to produce a heatmap representing fungal frequency in soil samples.

Additionally, the distribution and frequency of OTUs from selected fungal families were examined to identify those shared among sampling areas. Specifically, OTUs belonging to families previously reported in orchid-associated sites through metabarcoding analyses were selected. The matrix of presence/absence was thus plotted in R environment using the packages ‘tidyverse’ and ‘circlize’.

Orchid species richness (number of species) and total abundance (number of individuals) were compared against fungal OTU richness (number of families/genera) and relative abundance of genera to understand the association between orchid and fungal diversity matrix. Spearman correlations and linear regressions were performed to test associations between orchid and fungal diversity metrics.

## 3. Results

This study investigated how the fungal microbiome associated with orchids varies among these sites, with the aim of understanding mechanisms that could enhance meadow restoration in the future.

**Figure 2:**
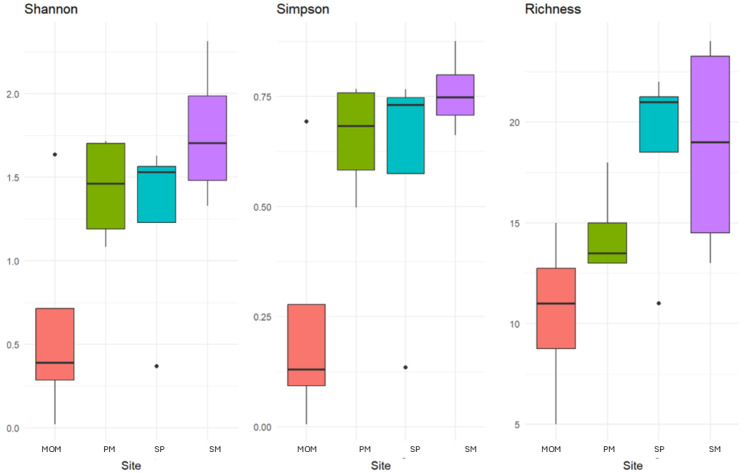
Shannon and Simpson indices and richness analysis of four sites at Essex Wildlife Trust, Grays Gorge (Sarsen Meadow (SM), Man Orchid Meadow (MOM), Penultimate Meadow (PM), and Sadgrove Plot (SP)) for both Ascomycota and Basdidiomycota fungal OTUs.

The Sadgrove Plot, situated within the gorge, exhibited the highest abundance of orchids among all surveyed locations. This site is characterized by exposed chalk and minimal vegetation, with very little grass cover, rendering it unimproved. Notably, it supports the highest abundance of key orchid-associated mycorrhizal fungi. In contrast, the three other sites, all located on the top shelf of the gorge, were established using chalk-rich soil transported from nearby areas. Alpha diversity values—calculated for all OTUs from both Ascomycota and Basidiomycota—varied among the sites, as indicated by the Shannon, Simpson, and species richness indices (Figure 2, Table 1 and Table S1). The Sarsen Meadow site exhibited the highest within-site diversity, whereas the Man Orchid Meadow recorded lower diversity values, particularly in the Shannon and Simpson indices. Despite these values, the Kruskal–Wallis test did not detect statistically significant differences in Shannon diversity among sites (χ^2^ = 1.875, df = 3, *p* = 0.5988). Post hoc comparisons with Dunn’s test and Benjamini–Hochberg correction confirmed the absence of significant pairwise differences between sites.

**Figure 3:**
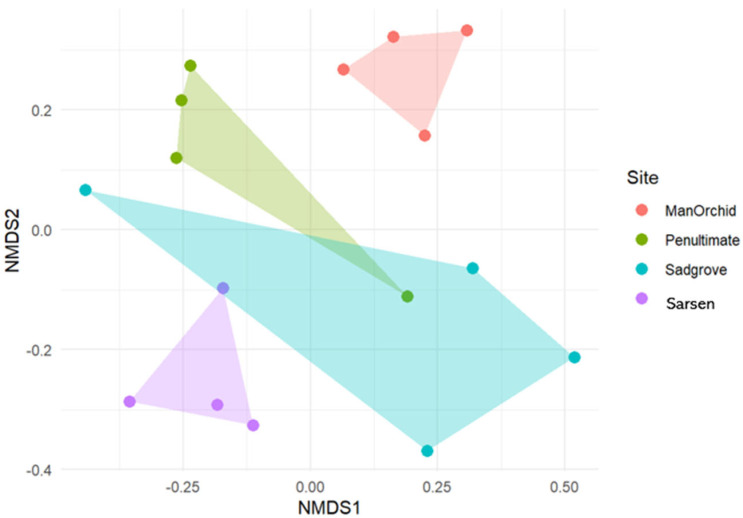
NMDS chord diagram showing distribution and overlap of selected Basidiomycota fungal families at Sarsen Meadow, Man Orchid Meadow, Penultimate Meadow, and Sadgrove Plot in Grays Gorge, Essex, with each arc corresponding either to a site or a fungal family, the connecting ribbons indicating shared associations based on OTU abundance/presence.

As for the Beta-Diversity analyses, the overall PERMANOVA showed a significant difference in OTU composition between sites (*p* = 0.001, R^2^ = 0.36). Pairwise analyses indicated that Sarsen Meadow was the most diverse, showing potentially significant differences with Man Orchid Meadow, Penultimate Meadow, and Sadgrove Plot. Sarsen Meadow exhibited the highest overall diversity, while Man Orchid Meadow showed the lowest for Basidiomycota fungi, especially mycorrhizal/mutualistic fungi (Figure 3). The visual representation via NMDS confirmed these trends (3D Stress = 0.11), with Sarsen Meadow clearly separated from the other sites, while Penultimate Meadow and Sadgrove Plot appeared more similar.

**Figure 4:**
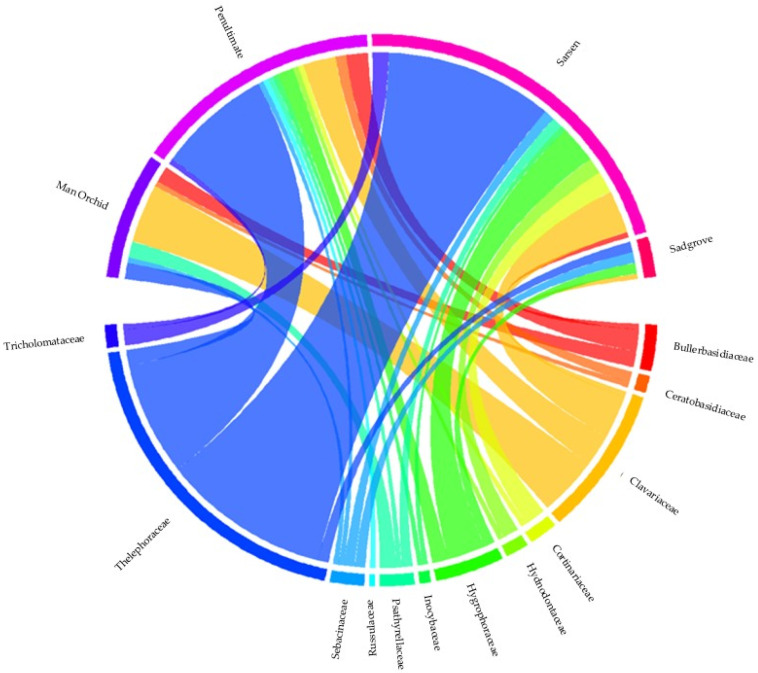
Heatmap showing distribution and frequency of occurrence of selected families of Basidiomycota at four orchid hosting sites at Sarsen Meadow, Man Orchid Meadow, Penultimate Meadow, and Sadgrove Plot in Grays Gorge, Essex.

The heatmap showing Spearman correlations between dominant fungal orders and the environmental gradient across sites. Fungal order tends to increase in abundance with more sparse or shrubby vegetation, showing a positive correlation (Figure S2) while fungal orders are more abundant in open or shaded grassland showing a negative correlation. Following the Kruskal–Wallis test, the *p*-values indicate whether the abundance of each fungal order differs significantly across sites (Table S2). Agaricales, Thelephorales, Sebacinales, and Glomerales showed significant differences. Cantherellales was missing from the top ten as the detectable amounts in soil are low but included in further analysis as showing in Figure S3. The heatmap presented in Figure 4 illustrates the distribution and frequency of selected OTUs from Basidiomycota, specifically those for which sequencing analyses permitted identification at the family level.

**Figure 5:**
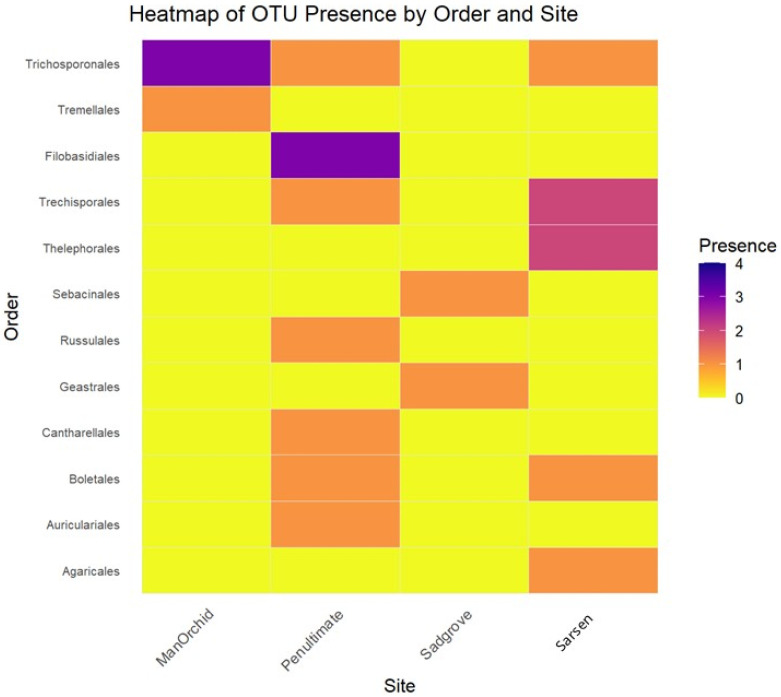
Heatmap showing relative abundance of key orders within Basidiomycota at Sarsen Meadow, Man Orchid Meadow, Penultimate Meadow, and Sadgrove Plot in Grays Gorge, Essex.

**Figure 6:**
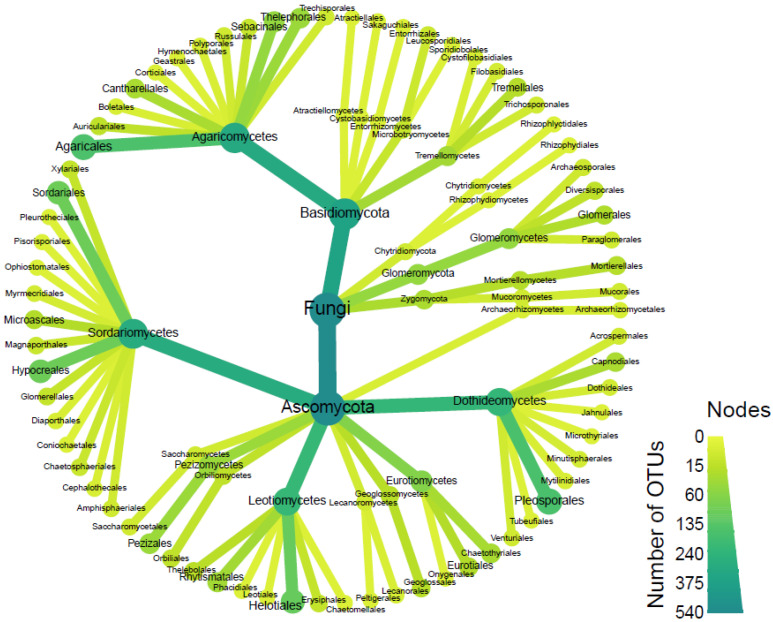
Total number of OTUs (including those genotypes not assigned to families) detected from Sarsen Meadow, Man Orchid Meadow, Penultimate Meadow, and Sadgrove Plot in Grays Gorge, Essex.

When data from all four sites were combined to evaluate the distribution and abundance of fungal orders, the resulting heatmap depicted the overall totals for the restored habitats at the Grays Gorge site in Essex. Among the Ascomycota, the order Pleosporales contained the highest number of OTUs. However, across all fungal orders, Agaricales exhibited the greatest number of OTUs (Figure 5 and Figure 6). Several orchid-associated mycorrhizal orders, including Cantharellales, Sebacinales, and Thelephorales, were represented by numerous OTUs.

**Figure 7:**
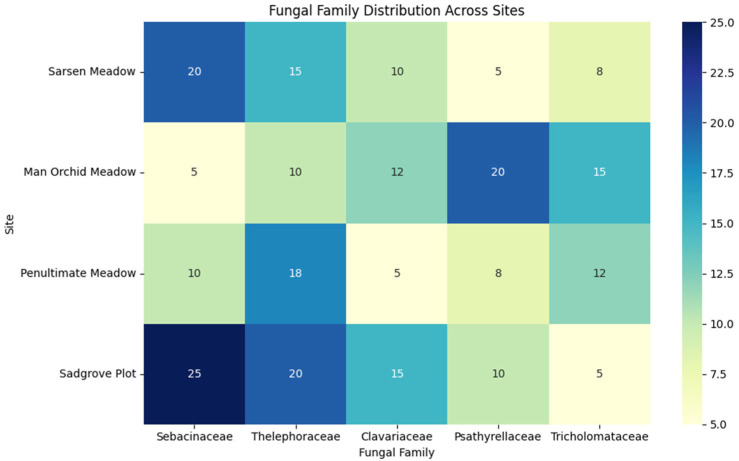
Mycorrhzial/mutualistic family distribution at Sarsen Meadow, Man Orchid Meadow, Penultimate Meadow, and Sadgrove Plot, with heatmap showing relative abundance of five key mycorrhizal fungal families.

Five fungal families Sebacinaceae, Thelephoraceae, Clavariaceae, Hygrophoraceae, and Psathyrellaceae, across the four meadow sites showed that Sadgrove Plot and Sarsen Meadow exhibit the highest diversity and abundance (Figure 7). A detailed analysis by including more mycorrhizal and mutualistic families show that Sadgrove Plot hosted more fungal families in greater abundance compared to other sites (Figure S1).

**Figure 8:**
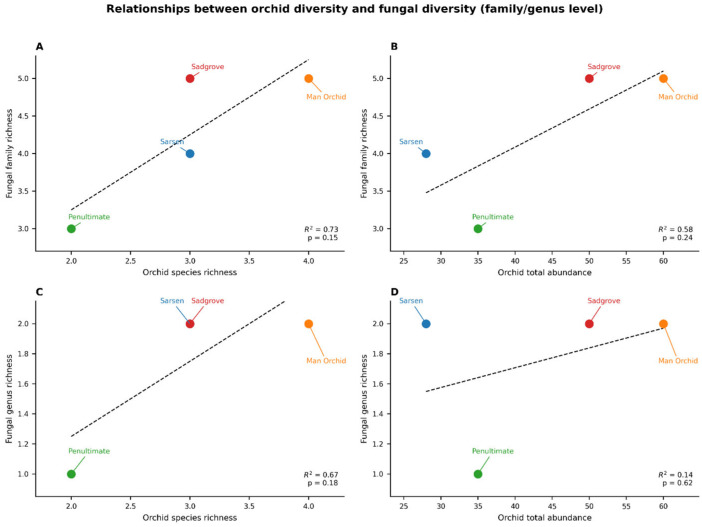
Relationships between orchid diversity and fungal diversity (family and genus levels) Sarsen Meadow, Man Orchid Meadow, Penultimate Meadow, and Sadgrove Plot. (**A**) Scatterplot showing the relationship between orchid species richness and fungal family richness, (**B**) Relationship between orchid total abundance and fungal family richness, (**C**) Relationship between orchid species richness and fungal genus richness, (**D**) Relationship between orchid total abundance and fungal genus richness. Dashed lines show regression fit with R^2^ and *p*-values.

Orchid diversity ranged from 2–4 species and 28–60 individuals per site while fungal diversity (family and genus) varied across sites, with some sites dominated by specific genera such as *Clavaria* which is not an orchid mycorrhizal fungus. Positive associations were observed between orchid abundance and fungal diversity at both family and genus levels. Regression models showed that orchid total abundance was a stronger predictor of fungal diversity than species richness (Figure 8).

**Figure 9:**
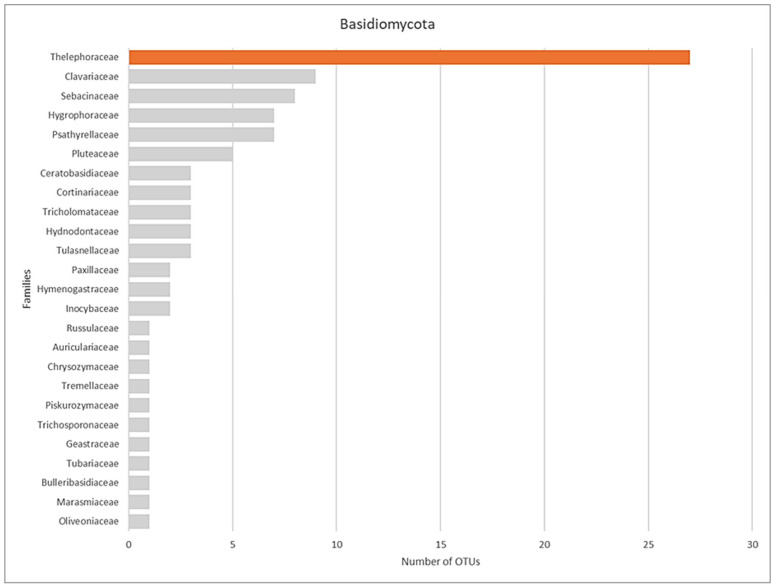
Number of OTUs of families from Basidiomycota at Sarsen Meadow, Man Orchid Meadow, Penultimate Meadow, and Sadgrove Plot in Grays Gorge, a former chalk quarry.

Family-wise comparison, of all sites, within Basidiomycota shows Thelephoraceae as the family with the greatest number of OTUs followed by Clavariaceae and Sebacinanceae (Figure 9). Regression analysis results support the hypothesis that both fungal abundance and diversity are higher near orchid colonies and decline with distance. Abundance trends in three meadows (Sarsen, Man Orchid, Penultimate) show steep negative slopes (1.8), meaning fungal abundance clearly decreases with distance. OTU diversity declines gently across all sites suggests this decline is consistent and predictable. The high R^2^ values across all graphs confirm that these patterns are statistically robust, even if the rate of decline varies by site (Figure 8 and Figure S4). Sadgrove Plot is the outlier with abundance declines only slightly (slope = 0.2), though the fit is still strong. However, Sadgrove Plot stands out across multiple families, for potentially known mycorrhizal families including Sebacinaceae (*Sebacina*) and Thelephoraceae (*Tomentella*) compared to the other sites. This pattern indicates higher fungal diversity and richness at Sadgrove, suggesting that its environmental conditions or host associations support a more complex fungal community (Supplementary Materials Figure S3). In contrast, Sarsen Meadow and Man Orchid Meadow show more restricted profiles, while Penultimate Meadow is intermediate.

**Figure 10:**
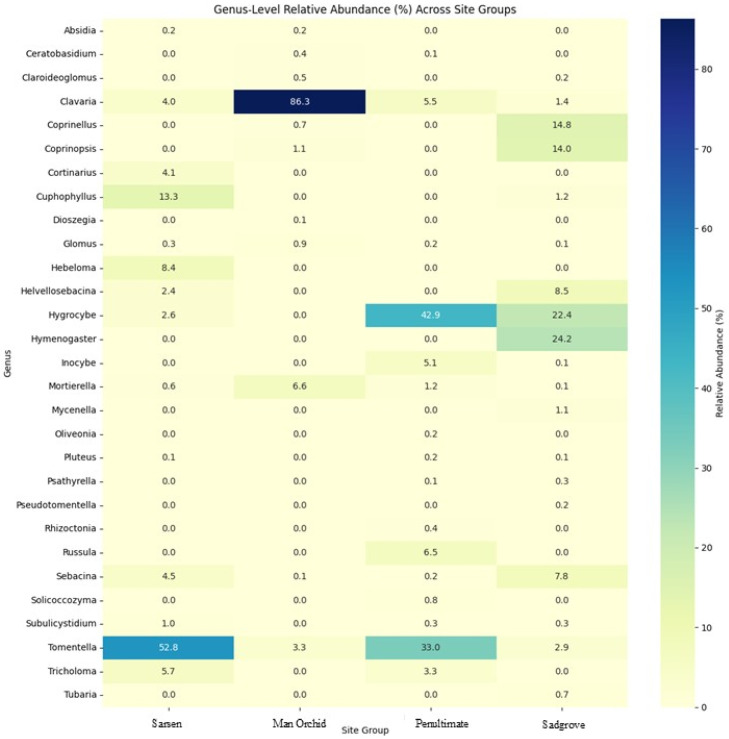
Average relative abundance and distribution of key genera of potential mycorrhizal/mutualistic fungi at Sarsen Meadow, Man Orchid Meadow, Penultimate Meadow, and Sadgrove Plot.

Figure 10 and Figure S5 displays the distribution and relative abundance of different key genera. *Clavaria*, *Tomentella*, and *Hygrocybe* are the most abundant genera of either mycorrhizal or mutualistic functions recorded from all sites. Of these, *Clavaria* was in very high abundance in the Man Orchid Meadow, while *Tomentella* was in high abundance in both Sarsen and Penultimate Meadows. Sadgrove Plot hosted *Hygrocybe* and *Hymenogaster* in relatively high abundance.

The soil fungal families Sebacinaceae and Thelephoraceae, which are known orchid mycorrhizal fungi, were analysed separately and the NMDS ordinations reveal distinct clustering patterns among sites for Thelephoraceae. This indicates strong compositional differences across locations, consistent with PERMANOVA results (*p* = 0.001 after Holm correction). In contrast, Sebacinaceae shows greater overlap among sites, suggesting a more uniform distribution of OTUs across the four habitats (Figure S6)

**Figure 11:**
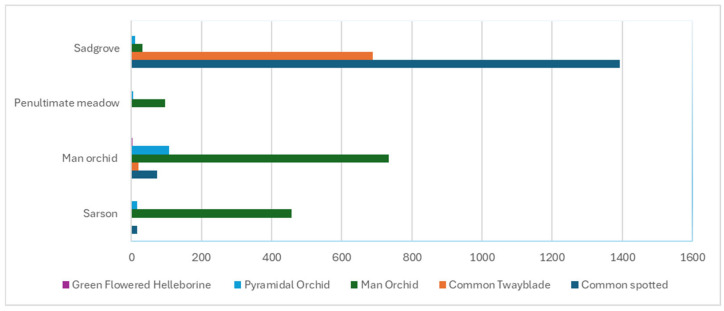
Diversity of orchids and number recorded in 2024 at Sarsen Meadow, Man Orchid Meadow, Penultimate Meadow, and Sadgrove Plot in Grays Gorge.

A comparative summary of orchid abundance, diversity, and fungal richness across sites showed that Sadgrove Plot had the highest orchid abundance and moderate diversity, while Sarsen Meadow had the highest fungal diversity (Figure 11, Table 2). Penultimate Meadow was the least diverse in both orchids and fungi.

## 4. Discussion

This study provides a comparative analysis of orchid habitats located on the top shelf and within the gorge of a restored chalk quarry. The top shelf, historically topped with imported soil, contrasts with the gorge base, which retains its original chalk substrate. These differences in substrate and management history have shaped the fungal communities and orchid colonization patterns observed across the four sites. The early autumn cut and collect, and the removal of shrubby vegetation happen on rotation, generally biannually. Of the more recently restored meadows, Sadgrove Plot (20 years) was cleared and topsoil stripped and Penultimate Meadow (10 years), was opened by removing the shrubs, which facilitated the appearance of orchids. These interventions significantly promoted the colonisation of orchids. The Sadgrove Plot exhibits the highest abundance of orchids, characterized by exposed chalk and minimal vegetation. Notably, it also supports the greatest abundance of key mycorrhizal fungi. The youngest meadow, Penultimate, demonstrates the lowest diversity and abundance of fungi. The Sarsen Meadow exhibited the highest within-site diversity, whereas the Man Orchid Meadow recorded lower diversity values, particularly in the Shannon and Simpson indices, although not statistically significant.

A study examining recent declines and range changes in selected European countries observed that most orchid populations declined in the northern part of the study area (specifically Northern France, Belgium, and Luxembourg), whereas many new occurrences were recorded in the central and southwestern regions of the study area [23]. A study of 42 European cities recorded very high diversity for orchids, with sand and clay pits hosting a number of rare orchids [14]. *Orchis anthropophora* has experienced a significant decline in Britain over the past century, and is currently both rare and classified as Endangered. The current sites studied are in the county of Essex, while the nearby county of Surrey has been recorded as hosting significant colonies across the full length of the chalk in the past [24]. Previously, it was reported that the current study sites had populations of several thousand plants [25]. In the neighbouring county of Kent, the species is reported as occurring in chalky soils in hedgerows, wood margins, rough grassland, quarries, and roadside verges [26]. The establishment of new populations is minimal, while many existing populations have become extinct, primarily due to agricultural intensification, such as the ploughing and fertilization of old fields [25]. Small populations have been recorded in many other southern counties in England since 1990 [27]. It is possible that the recruitment and establishment of new populations of *O. anthropophora* occurred in the gorge from the seed blown down from the top shelf. However, the mycorrhizal fungal groups that were found in the newly formed Penultimate Meadow and the 20-year-old meadow in Sadgrove Plot demonstrate the potential value of those fungi to support orchids of different types to colonise. The Man Orchid Meadow was the oldest area for man orchid and incidentally hosted a narrow diversity of mycorrhizal fungi. Mycorrhizal fungi of specific groups were found in the gorge and on the top shelf with detectable percentages. There may be other fungi of importance such as mycorrhizal fungi present, but not at the levels to be detected using the primer set used in this study.

When data from all four sites (three on the top shelf and one inside the gorge) were combined, Pleosporales (Ascomycota) exhibited the highest number of OTUs, while Agaricales (Basidiomycota) emerged as the order with the greatest number of OTUs, as reported before in grassland orchid habitats [28]. Soil from the most recently established meadow, Penultimate Meadow, had several fungal orders including, Filobasidiales, Russulales, Cantharellales, and Boletales, which have many of the mycorrhiza families. Less diverse in this regard is Sadgrove Plot, which lies within the gorge, and exhibited a relatively higher occurrence of OTUs belonging to the Trechisporales and Thelephorales orders. Older meadows such as Sarsen Meadow demonstrated the highest overall OTU diversity, while Man Orchid Meadow was found to be the least diverse. When Thelephoraceae and Sebacinaceae were analysed separately, Thelephoraceae was shown to exhibit site-specific associations or environmental filtering, whereas Sebacinaceae appears more generalist in its site occupancy. In a previous study, Tricholomataceae was a dominant fungus in the microhabitat of *Calanthe sieboldii* [29]. The majority of fungal families detected in this study were also found in rhizosphere soils in four *Cypripedium* spp., such as Psathyrellaceae, Russulaceae, Cortinariaceae, and Thelephoraceae [30].

*Neottia ovata* is considered one of the most common of all orchids and of Least Concern in Great Britain and Ireland, but it has declined by nearly 30% due to land use changes [31]. The gorge site, Sadgrove Plot, is found to be the best site for *N. ovata* compared to the other three sites on the top shelf. Flowering and seed set of *N. ovata* are affected by weather conditions, tending to decline during periods of rain but reaching optimal levels under dry conditions. The gorge formed because of decades of chalk mining and through targeted vegetation management still provides chalk and sparse vegetation even after 100 years of recovery. The Sadgrove Plot inside the gorge is prone to drying quickly due to the extremely thin or no topsoil and lack of vegetation cover compared to the sites on the top shelf. Nevertheless, this habitat is hosting unique fungal diversity compared to other sites on the top shelf, favouring a high density of common spotted and common twayblade, along with newly forming colonies of man and pyramidal orchids. A very high abundance of *Hymenogaster citrinus* was detected from the soil close to the man orchid populations in the gorge, but not from any other site. Hymenogatsaceae is an ectomycorrhizal group recorded to be associated with *Cephalanthera* spp. [32]. *Geodorum* and *Calanthe* have been found to have association with the genus *Coprinopsis* [33,34]. *Coprinopsis argentea* is detected in relatively high abundance within the chalky soil in the gorge. Sebacinales, reported as both endo and ectomycorrhizal, are ubiquitous fungi that are mutualistic with many plant groups, including orchids [35,36,37]. *Sebacina incrustans*, a mycorrhizal fungus, has been detected in very high abundance and recorded from areas where *N. ovata* colonises in abundance, but not detected from anywhere else. *Neottea nidus-avis*, a myco-heterotrophic orchid, was found to be critically dependent on *Sebacina dimitica* [38]. Some fungi are abundant in the rhizosphere soil, but they may not be critically important to the orchid. Further studies with the root microbiome are required to clarify this.

The studied sites are managed through an early autumn cut on rotation to maintain an open chalk grassland habitat, with the arisings collected and removed to prevent nutrient enrichment. This habitat management is essential for orchids to colonise and establish. There is an association between grassland management and fungi. Indeed, management to promote the diversity of grassland plants is also found to facilitate increased abundance of diverse grassland fungi such as waxcap fungi [39]. The most abundant mycorrhizal fungal groups in this study were Thelephoraceae and Sebacinaceae. Non-mycorrhizal Clavariaceae and potentially mutualistic and Hygrophoraceae, two fungal groups typical of seminatural grasslands, were also found to be abundant. Waxcaps, along with several other groups of fungi, such as the families Clavariaceae, share habitat requirements similar to those of many plant species typical of semi-natural grasslands [40].

The gorge site, Sadgrove Plot, hosted four species of orchids, the most of all study sites, with common spotted and common twayblade as the dominant taxa, and man orchid and pyramidal orchid increasingly colonising over the last few years. The focal orchid species in this study is the man orchid, which is endangered in Britain and is predominantly found in chalk and limestone grasslands and dune grasslands. They are found along field margins, abandoned chalk pits, limestone quarries, and roadside verges [25]. Members of the family non-mycorrhizal Clavariaceae are associated with nutrient-poor grasslands [39]; *Clavaria pullei* was detected at extremely high abundance from the Man Orchid Meadow but not at other sites, except for a small percentage from one of the samples from the Penultimate Meadow. No related lineage of this fungus is associated with orchids, but further studies on the root microbiome will help us understand whether they are an important genus for man orchid. Orchid mycorrhizal fungi are believed to be present in good abundance nearer the colonies and may tail off as the distance from the colonies decrease. A review by McCormick et al. on abundance of orchid mycorrhizal fungi with increased seed germination and protocorm development of few terrestrial orchids found a positive correlation [11,41,42].

This study builds upon two previous investigations from the same laboratory, which examined the soil microbiome in habitats hosting single orchid species such as *Dactylorhiza incarnata* ssp. *ochroleuca* and *Anacamptis morio* [17,28]. Expanding beyond single-species habitats, the current study explores orchid-rich landscapes with diverse soil characteristics and orchid assemblages. It highlights the potential benefits of understanding the relationships between soil fungal microbiomes and orchid diversity and abundance. Analysis to test association between orchid and fungal diversity matrix showed that, although in limited scale, the orchid abundance is found to be a robust indicator of fungal diversity, suggesting that denser orchid populations support richer fungal communities. Genus-level analysis revealed site-specific dominance of species like *Tomentella,* highlighting potential functional specificity in orchid–fungus associations. Dual-level fungal analysis (family and genus) provides complementary ecological insights with family-level patterns reflecting broad trends, while genus-level data revealing finer-scale specificity. Detailed studies with Tulasnella-specific primer and root microbiome are essential to explore this further. Conservation and restoration efforts should consider both orchid abundance and the presence of key fungal taxa to maintain ecosystem function and resilience. This is particularly important with regards to the resilience of orchid populations in the face of a changing climate.

## 5. Conclusions

To our knowledge this is the first of its kind in the UK exploring the importance of former quarries as future orchid-rich meadows. The study proved that former quarries can serve as optimal sites for establishing orchid meadows, based on the premise that higher orchid densities foster more diverse fungal communities. This study highlights how orchid colonization and fungal microbiome diversity in restored chalk quarry habitats are influenced by a combination of site history, substrate type, and management practices. Among the sites examined, Sadgrove Plot which is located within the gorge, supported the highest orchid abundance and a rich diversity of mycorrhizal fungi. This underscores the ecological importance of exposed chalk substrates and minimal vegetation cover.

When associations between orchid and fungal diversity matrices were tested, the analysis revealed that sites with higher orchid abundance tend to support greater fungal diversity, especially at the genus level, and certain fungal genera may play a role in orchid community structure. These insights are vital for refining conservation strategies and improving the ecological outcomes of grassland and quarry restoration projects.

Methodological limitations, including the use of a single primer set, likely led to underrepresentation of certain orchid-associated fungi. Future research should incorporate broader primer sets, seasonal sampling, and root-associated microbiome analyses to deepen our understanding of orchid–fungus interactions.

## Figures and Tables

**Table 1:** Table showing OTU composition of all fungi between sites with Permanova + Bonferroni correction at four orchid hosting sites in Essex Wildlife Trust, Grays Gorge.

	Comparison	F. Model	*R* ^2^	*p*-Value	*p*.adj
1	Sarsen vs. Man Orchid	4.250186	0.4146448	0.037	0.222
2	Sarsen vs. Penultimate	1.939709	0.2443048	0.027	0.162
3	Sarsen vs. Sadgrove	2.466139	0.2912944	0.027	0.162
4	Man Orchid vs. Penultimate	2.195664	0.2679056	0.107	0.642
5	Man Orchid vs. Sadgrove	2.449462	0.2898956	0.033	0.198
6	Penultimate vs. Sadgrove	1.050537	0.1490010	0.433	1.000

**Table 2:** Diversity and abundance of orchids and fungi at four meadows in Grays Gorge.

Site	Key Feature	Orchid Abundance	Orchid Diversity	Fungal Diversity
Sadgrove Plot	Gorge terrain, sparse vegetation	Highest	Moderate	Diverse (Agaricales dominant)
Sarsen Meadow	Top shelf; broad habitat variety	Moderate	Highest	Most diverse
Penultimate Meadow	Top shelf; open habitat, low vegetation	Lowest	Lowest	Limited diversity
Man Orchid Meadow	Top shelf; specialized microhabitat	Low	Low	Least diverse

## Data Availability

The original contributions presented in this study are included in the article and Supplementary Materials. Further inquiries can be directed to the corresponding author.

## Supplementary Materials

The following supporting information can be downloaded at: https://www.mdpi.com/article/10.3390/microorganisms13102390/s1. Figure S1. Sites annotated with their respective soil types and vegetation showing abundance of families from Basidiomycota at Sarsen Meadow, Man Orchid Meadow, Penultimate Meadow, and Sadgrove Plot; Figure S2. Heatmap showing Spearman correlations showing positive and negative correlations between dominant fungal orders at Sarsen Meadow, Man Orchid Meadow, Penultimate Meadow, and Sadgrove Plot; Figure S3. Distribution and relative abundance of key fungal orders from Basidiomycota Sarsen Meadow, Man Orchid Meadow, Penultimate Meadow, and Sadgrove Plot; Figure S4. Regression analysis of relative abundance and number of OTUs of Basidiomycota fungi at Sarsen Meadow, Man Orchid Meadow, Penultimate Meadow, and Sadgrove Plot in relation to large colonies of orchids and away from colonies; Figure S5. Heatmap showing log-transformed OTU detection frequencies for major fungal families and genera across Sarsen Meadow, Man Orchid Meadow, Penultimate Meadow, and Sadgrove Plot. Rows represent families and genera, and columns represent sites; Figure S6. Non-metric multidimensional scaling (NMDS) ordinations of fungal communities for Sebacinaceae and Thelephoraceae at Sarsen Meadow, Man Orchid Meadow, Penultimate Meadow, and Sadgrove Plot. Ordinations are based on Bray–Curtis dissimilarities of OTU abundances within each family. Points represent individual samples; focal site in each panel is shown in colour with a convex hull polygon, while other sites are displayed in light grey for context. Site names are printed inside hulls. Stress values for each family are shown in first panel of each row (Sebacinaceae: 0.199; Thelephoraceae: 0.244); Table S1. Shannon and Simpson indices and richness analysis of sites for both Ascomycota and Basdidiomycota fungal OTUs, showing comparisons within and between sites at Sarsen Meadow, Man Orchid Meadow, Penultimate Meadow, and Sadgrove Plot; Table S2. Kruskal–Wallis test showing *p*-values of key fungal orders at Sarsen Meadow, Man Orchid Meadow, Penultimate Meadow, and Sadgrove Plot.
