# Supplementary material for: Preliminary Assessment of Mycobiome at Former Quarry Site That Hosts a Diverse and Abundant Orchid Population
Source: Microorganisms. 2025 Oct 17;13(10):2390. doi: 10.3390/microorganisms13102390 (PMC12566108; doi:10.3390/microorganisms13102390)
Supplement: Supplementary file 1 [file microorganisms-13-02390-s001.zip › microorganisms-3882510-supplementary.pdf]

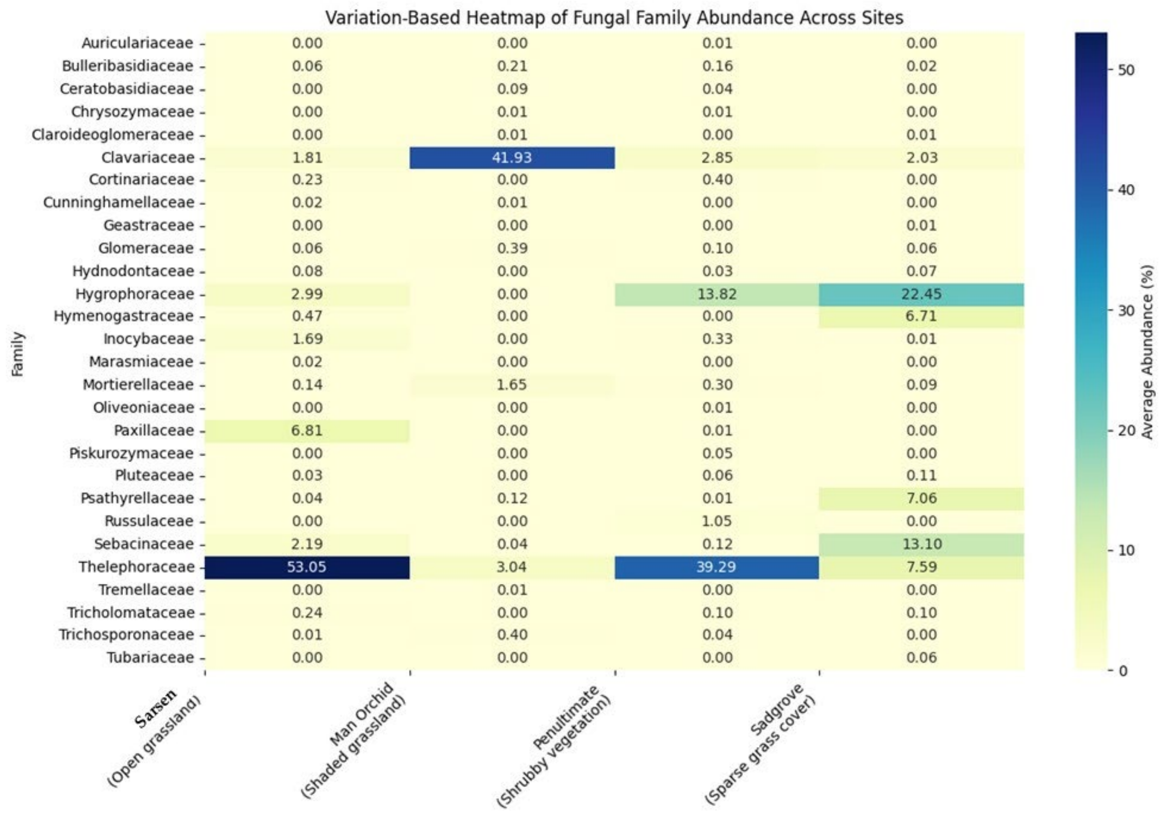

Figure S1. Sites annotated with their respective soil types and vegetation showing abundance of families from Basidiomycota at Sarsen Meadow, Man Orchid Meadow, Penultimate Meadow, and Sadgrove Plot.

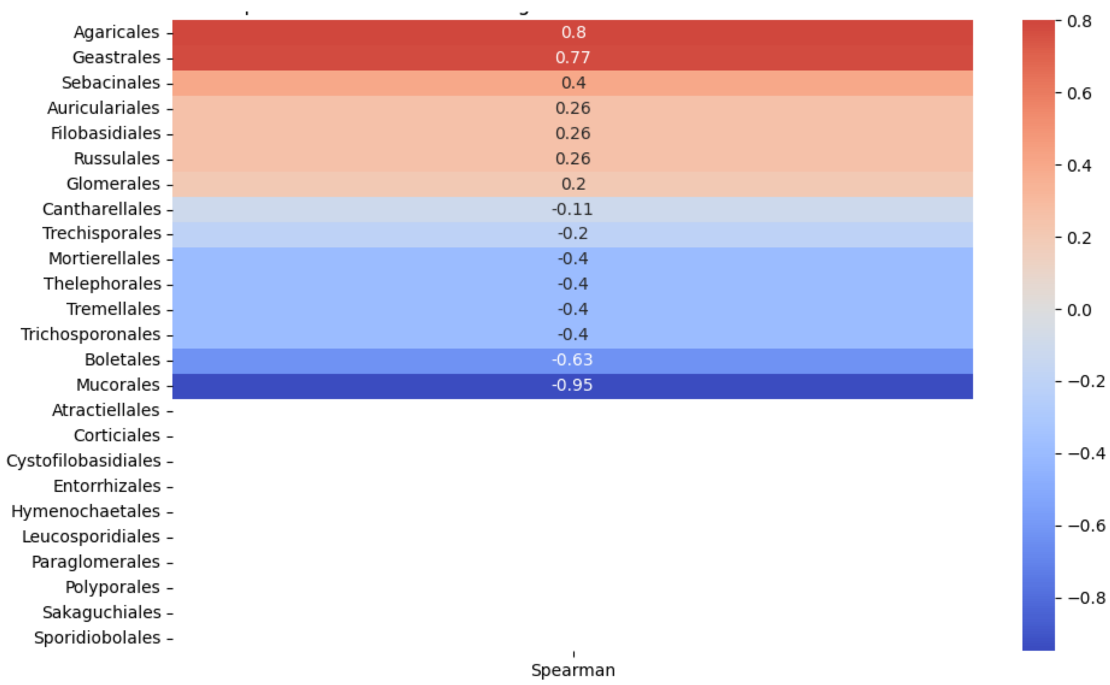

Figure S2. The heatmap showing Spearman correlations showing positive and negative correlations between dominant fungal orders at Sarsen Meadow, Man Orchid Meadow, Penultimate Meadow, and Sadgrove Plot.

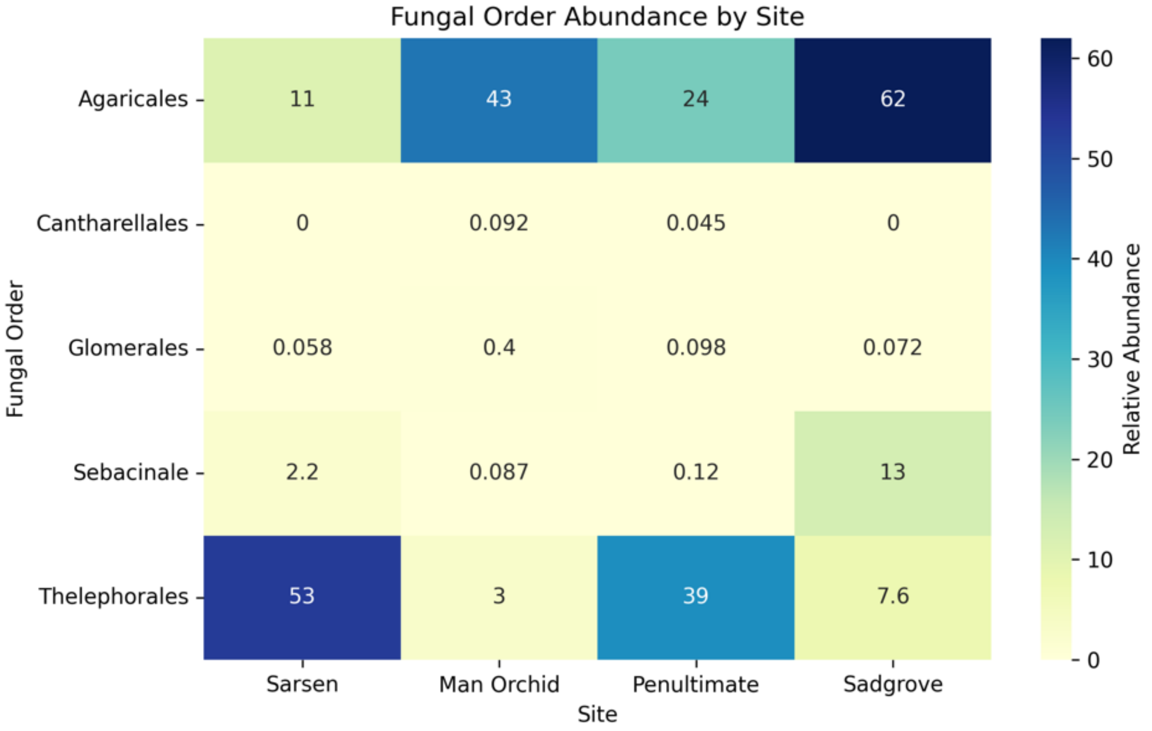

Figure S3. Distribution and relative abundance of key fungal orders at Sarsen Meadow, Man Orchid Meadow, Penultimate Meadow, and Sadgrove Plot.

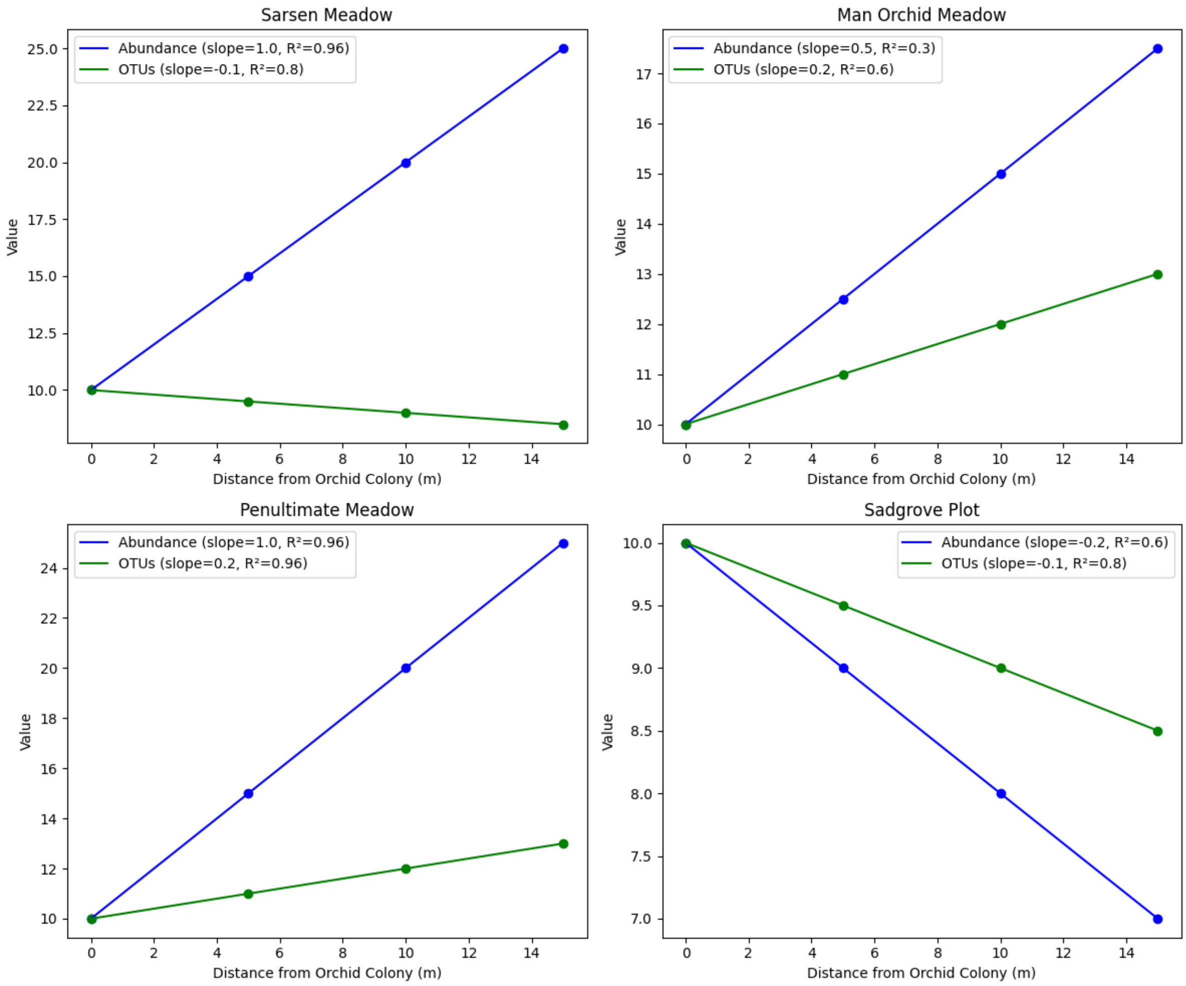

Figure S4. Regression analysis of relative abundance and number of OTUs of Basidiomycota fungi at Sarsen Meadow, Man Orchid Meadow, Penultimate Meadow, and Sadgrove Plot in relation to the large colonies of orchids and away from the colonies.

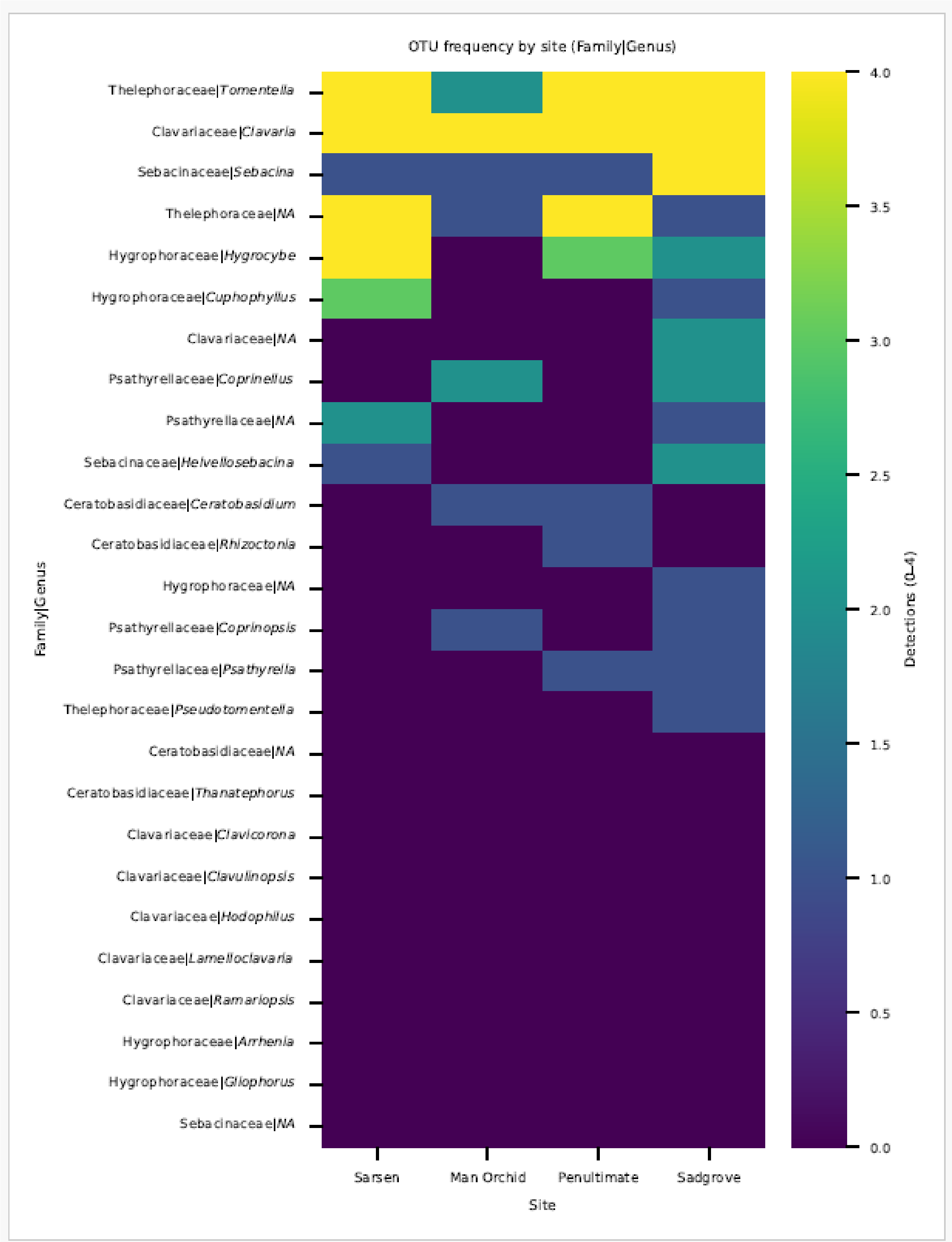

Figure S5. The heatmap shows log-transformed OTU detection frequencies for major fungal families and genera across Sarsen Meadow, Man Orchid Meadow, Penultimate Meadow, and Sadgrove Plot. Rows represent families and genera and columns represent sites.

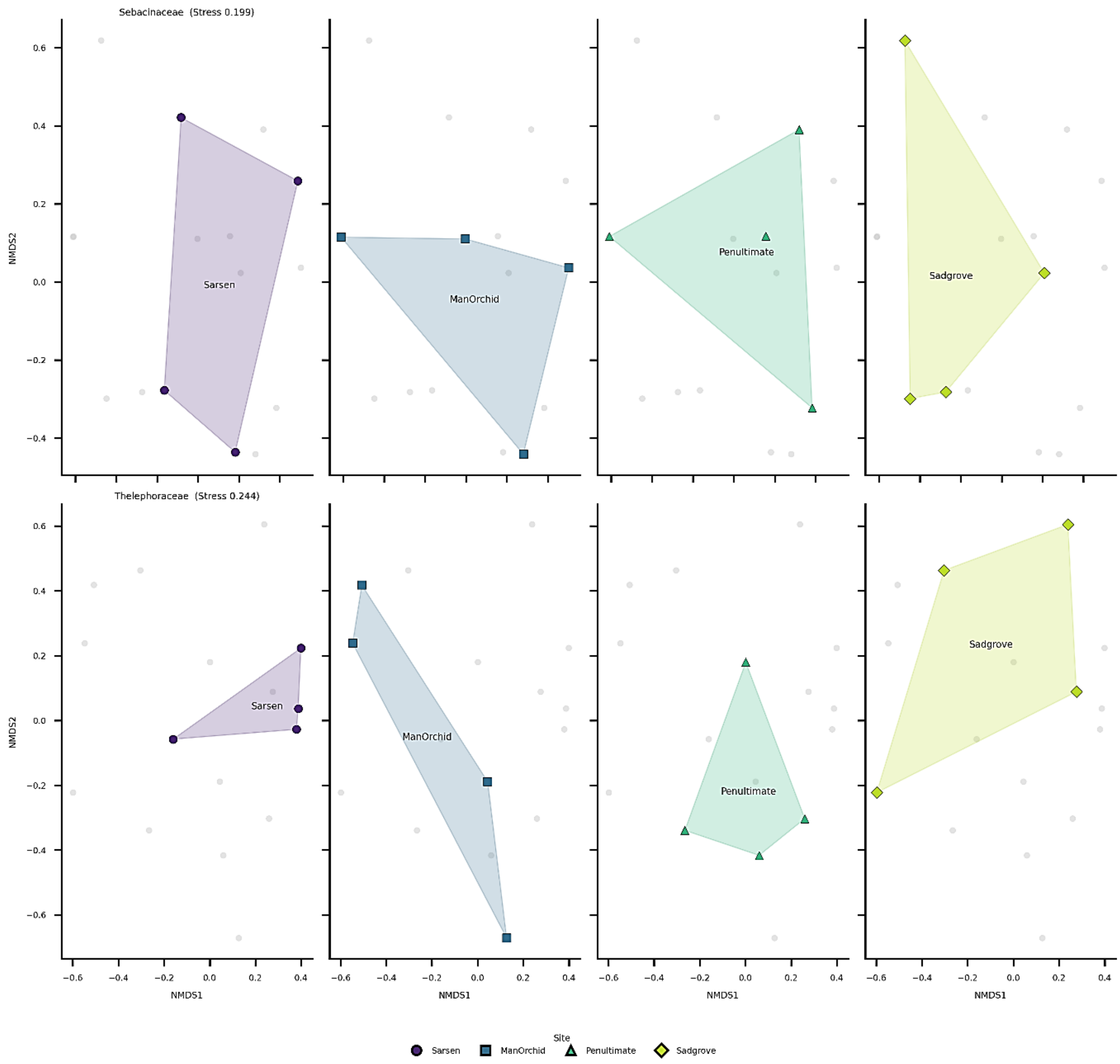

Figure S6. Non-metric multidimensional scaling (NMDS) ordinations of fungal communities for Sebacinaceae and Thelephoraceae at Sarsen, Man Orchid, Penultimate, and Sadgrove. Ordinations are based on Bray–Curtis dissimilarities of OTU abundances within each family. Points represent individual samples; the focal site in each panel is shown in colour with a convex hull polygon, while other sites are displayed in light grey for context. Site names are printed inside hulls. Stress values for each family are shown in the first panel of each row (Sebacinaceae: 0.199; Thelephoraceae: 0.244).

Table S1. Shannon and Simpson indices and Richness analysis of the sites for both Ascomycota and Basdidiomycota fungal OTUs showing comparisons within and between sites.

	Sample	Shannon	Site	Simpson	Richness
Sarsen1	Sarsen1	1.5328251	Sarsen	0.722061962	15
Sarsen2	Sarsen2	1.3278236	Sarsen	0.662613046	13
Sarsen3	Sarsen3	1.8764384	Sarsen	0.773629710	23
Sarsen4	Sarsen4	2.3123405	Sarsen	0.875166445	24
Man Orchid1	ManOrchid1	1.6361574	Man Orchid	0.692772010	10
Man Orchid2	ManOrchid2	0.3737203	Man Orchid	0.122102772	15
Man Orchid3	ManOrchid3	0.4082291	Man Orchid	0.138492191	12
Man Orchid4	ManOrchid4	0.0207853	Man Orchid	0.005068006	5
Penultimate1	Penultimate1	1.0819464	Penultimate	0.498212416	13
Penultimate2	Penultimate2	1.2258702	Penultimate	0.610663853	14
Penultimate3	Penultimate3	1.7171333	Penultimate	0.755155223	18
Penultimate4	Penultimate4	1.6954050	Penultimate	0.767392516	13
Sadgrove1	Sadgrove1	0.3693223	Sadgrove	0.134746391	11
Sadgrove2	Sadgrove2	1.5161026	Sadgrove	0.721806552	21
Sadgrove3	Sadgrove3	1.5433341	Sadgrove	0.740616740	21
Sadgrove4	Sadgrove4	1.6282082	Sadgrove	0.766227498	22

Table S2. Kruskal-Wallis Test showing the p-values of key fungal orders at at Sarsen Meadow, Man Orchid Meadow, Penultimate Meadow, and Sadgrove Plot.

Fungal Order	p-value	Interpretation
Agaricales	0.0044	Significant difference
Thelephorales	0.0138	Significant difference
Sebacinales	0.0219	Significant difference
Glomerales	0.0315	Significant difference
Tremellales	0.0905	Marginal difference
